# Genomic and phylogenetic characterization of human-adapted methicillin-resistant *Staphylococcus aureus* clonal complex 398 lineages in Taiwan

**DOI:** 10.1128/spectrum.02090-25

**Published:** 2025-12-23

**Authors:** Tsung-Hua Wu, Yung-Chieh Wu, Wen-Sheng Yeh, Mei-Hsiu Wan, Rou-Yi Li, Chun-Yi Lee, Yu-Ping Fang, Yu-Ying Yang, Yu-Fen Chang, Ying-Tsong Chen

**Affiliations:** 1 Doctoral Program in Medical Biotechnology, National Chung Hsing University 34916, Taichung, Taiwan; 2 Department of Pediatrics, Show Chwan Memorial Hospital 63295 , Changhua, Taiwan; 3 Department of Post Baccalaureate Medicine, College of Medicine, National Chung Hsing University 34916, Taichung, Taiwan; 4 Graduate Institute of Genomics and Bioinformatics, National Chung Hsing University 34916, Taichung, Taiwan; 5 Department of Pediatrics, Chang Bing Show Chwan Memorial Hospital, Changhua, Taiwan; 6 Graduate Institute of Chinese Medicine and Drug Development, National Chung Hsing University 34916, Taichung, Taiwan; 7 Department of Clinical Laboratory, Show Chwan Memorial Hospital 63295 , Changhua, Taiwan; 8 Department of Clinical Laboratory, Chang Bing Show Chwan Memorial Hospital, Changhua, Taiwan; 9 Institute of Molecular and Genomic Medicine, National Health Research Institutes, Maioli, Taiwan; Icahn School of Medicine at Mount Sinai, New York, New York, USA

**Keywords:** MRSA CC398, mobile genetic elements, phylogenetic analysis, ST1232, whole-genome sequencing

## Abstract

This study investigated the molecular epidemiology, genomic characteristics, and phylogenetic relationships of methicillin-resistant *Staphylococcus aureus* (MRSA) clonal complex 398 (CC398) isolates recovered from clinical infections in Taiwan. Fourteen CC398 MRSA isolates were identified between 2018 and 2022 from patients with skin and soft tissue infections and bloodstream infections at two regional hospitals. Whole-genome sequencing (WGS), core genome multilocus sequence typing, and comparative genomic analyzes revealed two distinct lineages: 10 sequence type (ST) 1232 and 4 ST398 isolates. Both ST1232 and ST398 carried SCC*mec* V; ST1232 carried SCC*mec* V(5C2), Panton–Valentine leukocidin (PVL), immune evasion cluster (IEC), and a *radC*::Tn*554* insertion. These isolates showed high genomic similarity to human-adapted CC398 strains from Japan and Korea. In contrast, ST398 isolates lacked PVL and Tn*554* but retained IEC genes, showing greater SCC*mec* diversity. None of the patients had livestock exposure, indicating possible community transmission. These findings highlight ST1232 as a clonally expanding, human-adapted MRSA lineage in Taiwan with distinct genetic traits and phylogenetic clustering with international isolates. Continued genomic surveillance is warranted to track its dissemination and guide infection control efforts.

**IMPORTANCE:**

This study reveals two human-adapted methicillin-resistant *Staphylococcus aureus* (MRSA) CC398 lineages in Taiwan: ST1232 and ST398. ST1232 carried Panton–Valentine leukocidin, immune evasion cluster (IEC), and *radC*::Tn*554*, while ST398 retained IEC and exhibited SCCmec variability. These findings highlight the public health importance of monitoring emerging MRSA lineages.

## INTRODUCTION

Methicillin-resistant *Staphylococcus aureus* (MRSA) remains a major global public health concern, causing skin and soft tissue infections (SSTIs), bloodstream infections (BSIs), and pneumonia (1). Among MRSA lineages, clonal complex 398 (CC398) has been predominantly associated with livestock and is recognized as a livestock-associated MRSA (LA-MRSA) lineage (2–5). Although originally linked to pigs and other animals, CC398 has demonstrated the ability to cross into humans and cause disease (4–7).

In recent years, however, a distinct human-adapted variant of CC398 has emerged in Asia and Australasia, characterized by the acquisition of mobile genetic elements (MGEs), such as the immune evasion cluster (IEC) carried on φSa3 prophages and the Panton–Valentine leukocidin (PVL) phage φSa2 (8–14). These elements are considered critical markers of human adaptation and are frequently observed in sequence type (ST) 1232, a single-locus variant of ST398 increasingly reported in Japan, Korea, and other regions (11–14). Comparative genomic studies have shown that human-adapted CC398 strains differ from livestock-associated ones not only in their virulence determinants but also in antimicrobial resistance profiles and evolutionary trajectories (14–17).

In Taiwan, knowledge of MRSA CC398 remains limited. Earlier molecular surveillance identified ST398 colonization in nursing homes (18), and a recent pediatric infection caused by PVL-positive ST1232 underscored its potential clinical relevance (19). Nonetheless, genomic data on Taiwanese CC398 isolates, particularly ST1232, are scarce.

This study aims to characterize CC398 MRSA isolates collected from two regional hospitals in central Taiwan between 2018 and 2022. Using whole-genome sequencing (WGS) and comparative genomic analysis, we investigated the genetic diversity, antimicrobial resistance, and mobile genetic elements of ST398 and ST1232 isolates. We further integrated these data with global CC398 genomes to place the Taiwanese isolates into their broader phylogenetic context.

## MATERIALS AND METHODS

### Study design and bacterial isolates

This study analyzed MRSA isolates collected from two sources: pus and wound swabs from patients with SSTIs in 2018, and blood cultures from patients with BSIs between 2018 and 2022. All non-duplicate MRSA isolates obtained during routine clinical diagnostics at two regional hospitals in central Taiwan—Show Chwan Memorial Hospital and Chang Bing Show Chwan Memorial Hospital—were subjected to multilocus sequence typing (MLST), and those identified as CC398 were selected for WGS.

For SSTI-derived isolates, bacterial colonies grown on culture media were first screened by coagulase testing, and species identification as well as methicillin resistance determination were confirmed using the BD Phoenix 100 automated system with the PMIC/ID-95 panel, following Clinical and Laboratory Standards Institute (CLSI) guidelines (20). For BSI isolates, blood samples were incubated in the BD BACTEC FX system, and organisms recovered from positive bottles were subsequently identified and confirmed as MRSA using the BD Phoenix 100 with the PMIC/ID-95 panel. Clinical data, including infection type, patient history, and clinical outcomes, were retrieved from electronic medical records when available.

### Sequence typing (MLST)

Genomic DNA was extracted using the QIAamp Blood DNA Mini Kit (Qiagen, USA) following the manufacturer’s instructions and stored at −80°C until analysis. MLST was performed based on the sequences of seven housekeeping genes of *S. aureus* (*arc*, *aroE*, *glp*, *gmk*, *pta*, *tpi*, and *yqiL*), as previously described (21), and allele profiles were assigned using the MLST database (https://pubmlst.org/organisms/staphylococcus-aureus).

### Antimicrobial susceptibility testing

Antimicrobial susceptibility was assessed using the BD Phoenix 100 Automated Microbiology System PMIC/ID-95, which includes 14 antibiotics: clindamycin, doxycycline, daptomycin, erythromycin, fusidic acid, ciprofloxacin, levofloxacin, linezolid, oxacillin, penicillin, trimethoprim-sulfamethoxazole, tetracycline, teicoplanin, and vancomycin. The interpretation of susceptibility results followed the CLSI guidelines (20).

### WGS, assembly, annotation, and sequence analyses

Genomic DNA was extracted using the DNeasy Blood & Tissue Kit (QIAGEN) and quantified with a Qubit Fluorometer. WGS was performed using both long-read (MinION, Oxford Nanopore Technologies) and short-read (iSeq 100, Illumina) platforms. For long-read sequencing, libraries were prepared using the Rapid Barcoding Sequencing Kit and sequenced on R9.4 or R10.4 flow cells. For short-read sequencing, libraries were prepared using the Nextera DNA Prep Kit and sequenced with 150 bp paired-end on an iSeq 100 (Illumina).

Genome assembly was performed using hybrid and long-read-only approaches, employing Unicycler v0.4.8 and CulebrONT v2.2.0 pipeline (configured with Flye as the long-read assembler) using R9.4 and R10.4 data. Assembly quality was assessed using QUAST and BUSCO (22, 23), and genome annotation was performed using the NCBI Prokaryotic Genome Annotation Pipeline (24). MLST, SCC*mec* typing, and *spa* typing were conducted using MLST v2.0, SCCmecFinder v1.2, and spaTyper, respectively (25–27).

Comparative genomic analyses were conducted using Mauve for global alignment and BLAST Ring Image Generator (BRIG) for visualization. Antimicrobial resistance genes, virulence factors, and prophages were identified using CARD, VFanalyzer, and PHASTER (28–32). Transposons and insertion sequences were annotated using TnCentral (33), and pairwise sequence comparisons were conducted using the NCBI BLASTn.

### Core-genome MLST and phylogeny

A phylogenetic tree based on cgMLST was constructed using Parsnp v2.0.5 (34), with *S. aureus* strain M2009_10004208 (GenBank accession no. SAMN00811588) as the outgroup. Genomic sequences used for phylogenetic analysis were retrieved from the NCBI GenBank database (Tables S1 and S2). The tree was visualized using FigTree v1.4.4 (http://tree.bio.ed.ac.uk/software/figtree/).

## RESULTS

### Prevalence and clinical characteristics of CC398 MRSA isolates

A total of 542 MRSA isolates were collected in this study, including 219 from SSTIs in 2018 and 323 from BSIs between 2018 and 2022. Out of 542 isolates, 14 isolates (2.6%) were identified as CC398 MRSA by MLST, comprising 4 from SSTIs and 10 from BSIs. These CC398 isolates belonged to three genotypes: ST1232-V (5C2) (*n* = 10), ST398-V (5C2&5) (*n* = 3), and ST398-V (5C2) (*n* = 1). The patients ranged in age from 6 months to 92 years, and none had a documented history of livestock contact. Notably, three BSI cases resulted in fatal outcomes.

In terms of antimicrobial susceptibility, ST1232 isolates exhibited a multidrug-resistant phenotype, uniformly resistant to clindamycin, erythromycin, and tetracycline (Table 1). In contrast, the four ST398 isolates were generally more susceptible: all were susceptible to tetracycline, and only one isolate was resistant to clindamycin and erythromycin. However, resistance to trimethoprim-sulfamethoxazole was observed in three of the four ST398 isolates.

**TABLE 1:** Molecular characteristics of CC398 MRSA isolates and their antibiotic resistance profiles^*a*^

Strain	Year	Sex	Age (yrs)	Source	Diagnosis	Skin pus MRSA	30-day outcome	MRSA clone	*spa* type	CC	E	LEV	SXT	OX	P	TE	VA
CBTW2018367	2018	Male	27	Skin pus	Cutaneous abscess	Yes	Survived	ST1232-V (5C2)	t571	R	R	S	S	R	R	R	S
CBTW2018008	2018	Male	49	Skin pus	Furuncle	Yes	Survived	ST1232-V (5C2)	t34	R	R	S	S	R	R	R	S
CBTW2018043	2018	Male	3	Skin pus	Cellulitis	Yes	Survived	ST1232-V (5C2)	t34	R	R	S	S	R	R	R	S
CBTW2018311	2018	Female	22	Skin pus	Sebaceous cyst	Yes	Survived	ST1232-V (5C2)	t34	R	R	S	S	R	R	R	S
SCTW2018482	2018	Male	55	Blood	Cellulitis	Yes	Survived	ST1232-V (5C2)	t34	R	R	S	S	R	R	R	S
SCTW2018694	2018	Female	84	Blood	Necrotizing abscess	Yes	Survived	ST1232-V (5C2)	t34	R	R	S	S	R	R	R	S
CBTW20181217	2018	Male	36	Blood	Cellulitis	Yes	Survived	ST1232-V (5C2)	t34	R	R	S	S	R	R	R	S
SCTW2018065	2018	Female	92	Blood	Pneumonia	Negative	Died	ST398-V (5C2)	t34	S	S	S	S	R	R	S	S
SCTW2019099	2019	Male	54	Blood	Cellulitis	Yes	Survived	ST398-V (5C2&5)	t34	R	R	S	R	R	R	S	S
SCTW2019145	2019	Male	62	Blood	Cellulitis	Yes	Died	ST398-V (5C2&5)	t34	S	S	S	R	R	R	S	S
SCTW2021015	2021	Male	82	Blood	Pneumonia	Negative	Died	ST398-V (5C2&5)	t571	S	S	S	R	R	R	S	S
CBTW2022135	2022	Male	0.5	Blood	Mediastinal abscess	Yes	Survived	ST1232-V (5C2)	t34	R	R	S	S	R	R	R	S
CBTW2022289	2022	Male	52	Blood	Necrotizing fasciitis	Yes	Survived	ST1232-V (5C2)	t34	R	R	S	S	R	R	R	S
SCTW2022336	2023	Female	67	Blood	Osteomyelitis	Yes	Survived	ST1232-V (5C2)	t34	R	R	S	S	R	R	R	S

^*a*^ CC, clindamycin; E, erythromycin; LEV, levofloxacin; OX, oxacillin; P, penicillin; SXT, trimethoprim-sulfamethoxazole; TE, tetracycline; VA, vancomycin; R, resistant; S, susceptible.

### Genomic comparison of CC398 MRSA chromosomes

**Fig 1:**
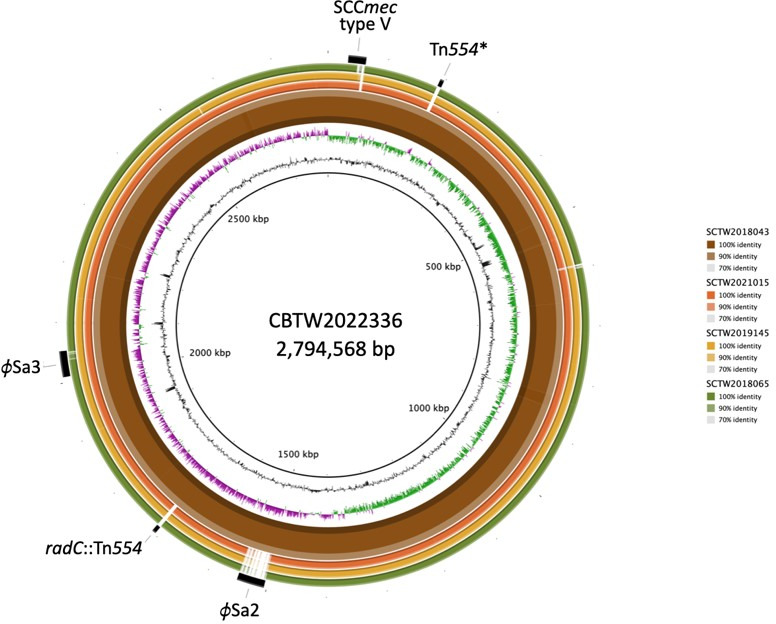
Comparative genomic analysis of CC398 MRSA isolates based on chromosomal content. A circular genome map was generated using BRIG, with CBTW2022336 (an ST1232 isolate) as the reference genome. The three outermost colored rings represent three ST398 isolates, while the thick composite colored ring is SCTW2018043, which represents all the highly conserved ST1232 group. Gaps in the colored rings indicate regions absent or highly divergent compared with the reference genome. The innermost rings display GC content and GC skew of the reference. Key mobile genetic elements—including SCC*mec* V, Tn*554*, and prophages φSa2 and φSa3—are indicated by black annotations on the outermost ring. The Tn*554* element inserted at the *radC* locus in ST1232 isolates is labeled as *radC*::Tn*554*, while a separate Tn*554* located elsewhere in the reference genome is labeled as Tn554*.

Whole-genome shotgun sequencing, assembly, and annotation were performed for the CC398 MRSA isolates. Comparative genomic analysis based on chromosomal content revealed a clear separation of these isolates into two distinct groups, ST1232 and ST398, corresponding precisely to their MLST-defined sequence types. This grouping was driven by differences in multiple chromosomal regions associated with MGEs, as illustrated in the BRIG-generated circular genome map (Fig. 1), using CBTW2022336, an ST1232 isolate sequenced in this study, as the reference genome.

The ST1232 isolates exhibited high overall sequence similarity and strong genomic conservation, while the ST398 isolates displayed greater variability, particularly in genomic regions harboring prophages, transposons, and other MGEs. SCC*mec* V was consistently identified in both ST1232 and ST398 isolates, representing a shared MGE element. Tn*554* transposons were also present across all genomes; however, a specific insertion of Tn*554* at the *radC* chromosomal locus (*radC*::Tn*554*) was found exclusively in ST1232 isolates but absent in the corresponding region of ST398.

In addition, prophage φSa2 was detected only in ST1232 isolates, whereas prophage φSa3 was present in both lineages, suggesting it is conserved across the CC398 clade. As illustrated in Fig. 1, this genome-wide comparison provides an overview of the chromosomal differences between ST1232 and ST398, highlighting lineage-specific features, such as *radC*::Tn*554* and prophage elements.

### Phylogenetic analysis and population structure

**Fig 2:**
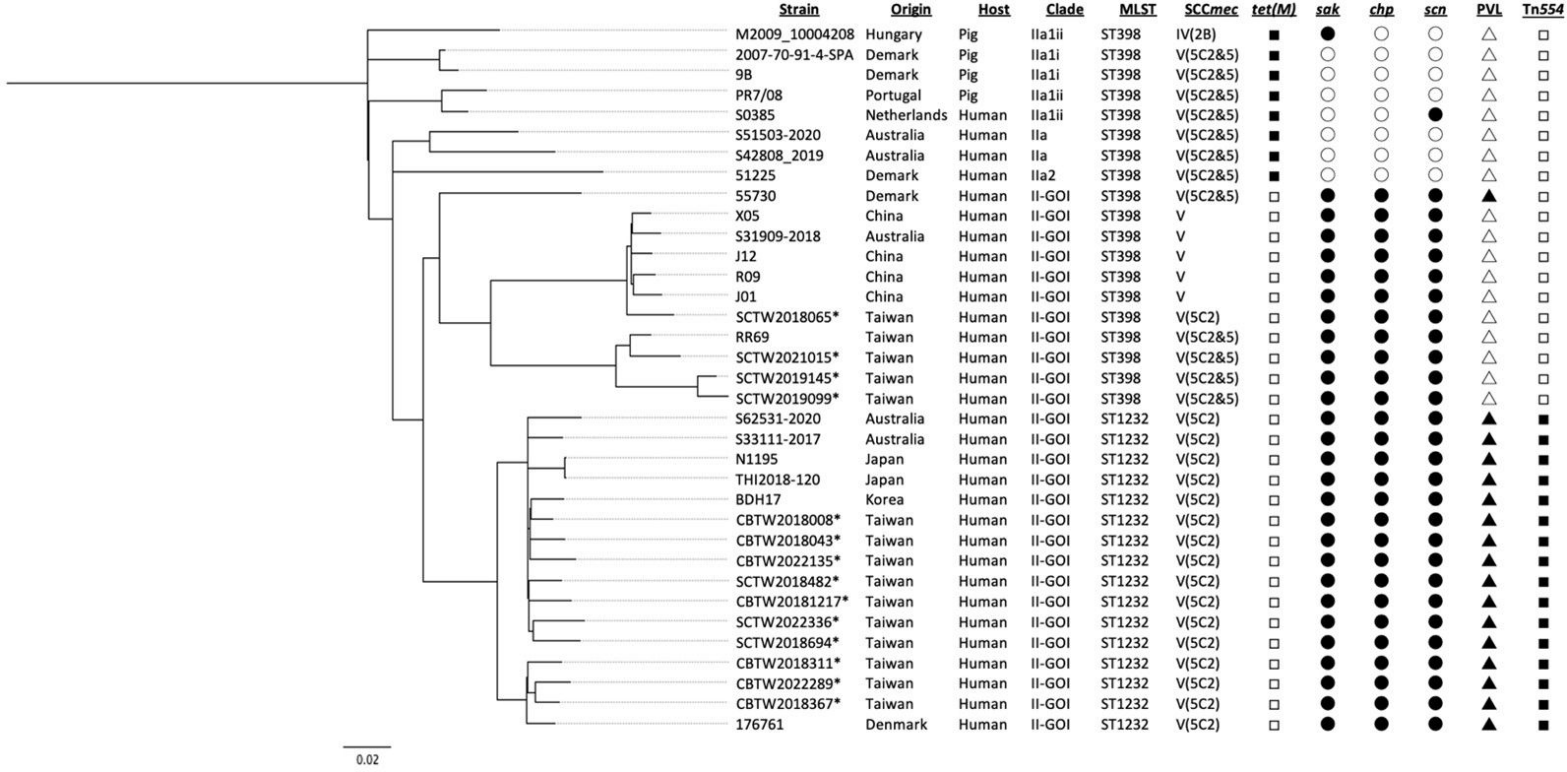
Core-genome phylogeny of MRSA CC398 isolates from Taiwan (2018–2022) and 21 reference genomes from public databases (Table S1). The phylogenetic tree was constructed using cgMLST with Parsnp v2.0.5 and rooted with strain S0385. Each isolate is annotated with its MLST type, SCC*mec* type, and a panel of genetic markers displayed as shape-coded symbols to the right of the tree. Filled symbols indicate the presence of a marker; empty symbols indicate its absence. Isolates are labeled by host, country, and clade, as defined in previous studies. Asterisks denote Taiwanese isolates from this study. The scale bar represents the number of nucleotide substitutions per site across the ~2.5 Mb core genome alignment; the 0.02 scale bar shown in the figure corresponds to ~50,000 SNPs. MRSA, methicillin-resistant *Staphylococcus aureus*; CC, clonal complex; ST, sequence type; MLST, multilocus sequence typing; SCC*mec*, staphylococcal cassette chromosome *mec*; PVL, Panton–Valentine leukocidin.

We performed cgMLST using WGS from 14 Taiwanese CC398 MRSA isolates, along with publicly available genomes representing global CC398 lineages. This global comparison placed the Taiwanese isolates within the broader phylogenetic landscape of MRSA and allowed assessment of their evolutionary relationships with known international lineages. The combined data set was used to construct a phylogenetic tree that revealed distinct clustering patterns (Fig. 2; Table S1). Each isolate was annotated with its MLST type, SCC*mec* type, and the presence or absence of key genetic markers—including *ask*, *cap*, *scn*, PVL, and *radC*::Tn*554*.

Consistent with previous reports (14), the isolates grouped into three major clades: IIa, PVL-negative II-GOI (group of interest), and PVL-positive II-GOI. The IIa clade, which includes LA-MRSA, was characterized by the absence of IEC genes and the presence of *tet(M)*. Four Taiwanese ST398-V isolates clustered within the PVL-negative II-GOI clade, consistent with human-adapted characteristics defined by the presence of IEC genes, while the uniform carriage of SCC*mec* V represents a lineage-associated feature commonly observed in community-associated MRSA. Among them, SCTW2021015 was closely related to the previously reported Taiwanese isolate RR69, and SCTW2019145 and SCTW2019099 also grouped within the same subclade. In contrast, SCTW2018065 was placed on a distinct branch, phylogenetically closer to Chinese human-associated ST398 strains (X05, J12, and R09).

The 10 Taiwanese ST1232-V (5C2) isolates clustered within the PVL-positive II-GOI clade, together with isolates from Denmark, Australia, Korea, and Japan. All ST1232 isolates carried IEC genes, *blaZ*, and *mecA* and consistently harbored *erm(A)* and *tet(K)*. In these isolates, *erm(A)* was located on the Tn*554* transposon, and the *radC*::Tn*554* insertion was observed only in this lineage. Consistent with these conserved genomic features, pairwise SNP analysis revealed differences ranging from 71 to 795 SNPs (mean, 350), indicating that the isolates are closely related but not genetically identical. Notably, even the maximum intra-lineage divergence (795 SNPs) was much smaller than the several thousand SNPs separating ST1232 from other CC398 clades, underscoring the high genetic homogeneity of this lineage despite limited microevolution.

### SCC*mec* typing and structural variability

Building on the results of WGS, BRIG visualization, and cgMLST analysis, structural differences in the SCC*mec* regions among CC398 MRSA isolates were further investigated. Pairwise sequence comparisons were performed using the NCBI BLAST tool, focusing on the chromosomal SCC*mec* loci. Fourteen Taiwanese CC398 MRSA isolates (4 ST398 and 10 ST1232) were analyzed alongside strains from Australia (S33111-2017, S62531-2020), Korea (BDH17), Japan (TH12018-120, N1195), and Denmark (176761). For these strains, only draft genomes with contig-level assemblies were available.

**Fig 3:**
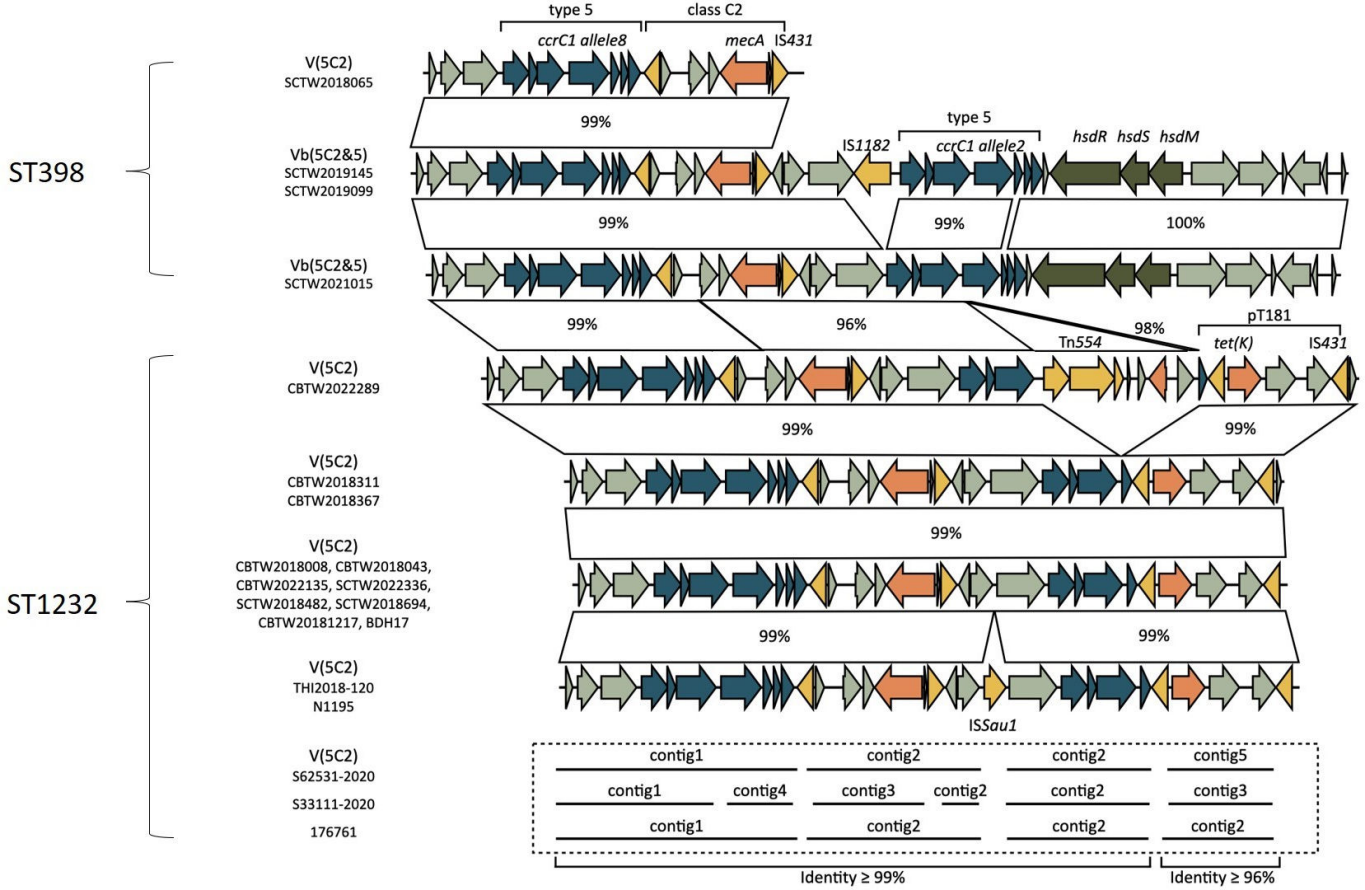
Schematic comparison of SCC*mec* V elements among ST398 and ST1232 MRSA isolates, based on BLASTn analysis. Genes are depicted as arrows, with orientation indicating the direction of transcription. Representative SCC*mec* structures from both ST398 and ST1232 isolates are shown. Gene annotations were assigned based on sequence homology using BLAST. Allelic variation was observed in *ccrC1* (allele 2 vs allele 8), along with differential integration of insertion sequences such as IS*1182*, IS*431*, and IS*Sau1*. The presence of Tn*554* and *tet(K)* was also noted in selected isolates. For isolates with incomplete SCC*mec* regions, contig-based assemblies are shown.

Substantial structural variation was observed among the ST398 isolates. The size of their SCC*mec* elements ranged from approximately 16 to 40 kbp, and differences were noted in *ccrC1* allele types (allele 2 vs allele 8), the arrangement and presence of restriction-modification system genes (*hsdR*, *hsdS*, *hsdM*), and the integration of mobile elements, such as IS*1182* and IS*431*. These components were variably present (Fig. 3). In contrast, SCC*mec* elements in ST1232 isolates were structurally conserved, all classified as type V (5C2) with lengths of approximately 30 kbp and ≥99% sequence identity across most regions. The *ccrC1* allele 8, *mecA*, and core backbone genes were consistently present. Minor variations were detected in certain strains—for example, isolate CBTW2022289 uniquely carried a Tn*554* element integrated within the SCC*mec* region, while others exhibited integration of IS*Sau1* (Fig. 3).

### Mobile genetic elements and resistance genes

**Fig 4:**
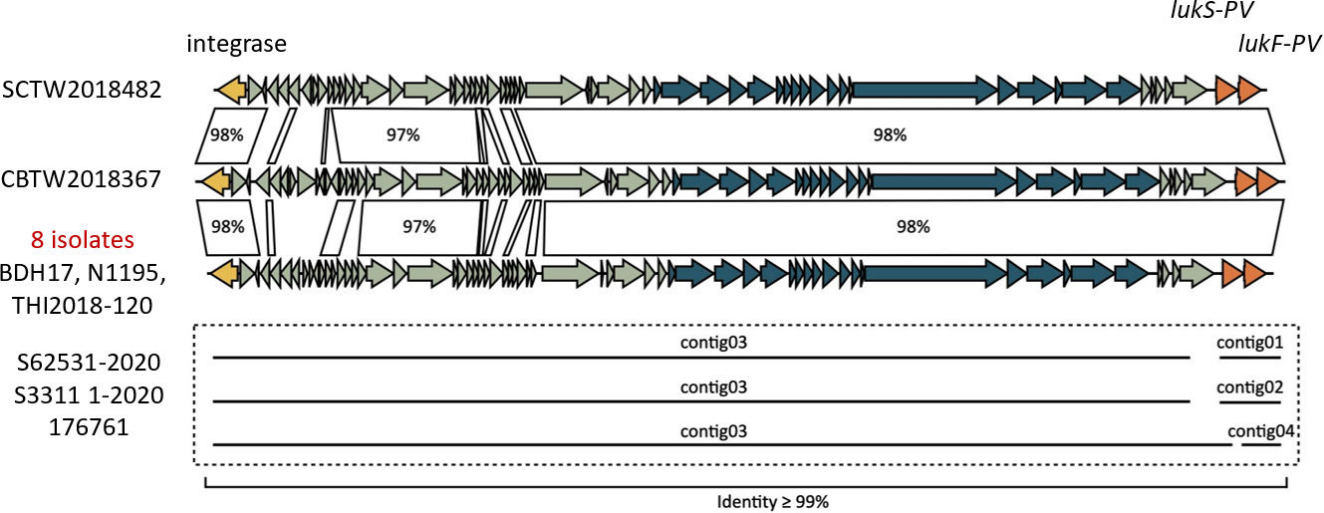
Schematic comparison of PVL-encoding prophages (φSa2) from representative MRSA ST1232 isolates, based on BLASTn analysis. The diagram includes 10 Taiwanese isolates (SCTW2018482, CBTW2018367, CBTW2018008, CBTW2018043, CBTW2018311, SCTW2018694, CBTW20181217, CBTW2022135, CBTW2022289, and SCTW2022336) and six international isolates from Korea (BDH17), Japan (TH12018-120 and N1195), Australia (S33111-2020, S62531-2020), and Denmark (176761). Genes are shown as arrows indicating transcriptional orientation and annotated based on sequence homology. The PVL toxin genes *lukS-PV* and *lukF-PV*, located at the right end of each prophage, are highlighted. Despite some assemblies being contig-based, all prophages showed high structural conservation (≥97–99% sequence identity) across the entire region.

In addition to SCC*mec*, we investigated the distribution and conservation of other MGEs, including prophages and transposons, and their associated resistance and virulence genes among CC398 isolates. To assess the conservation of PVL-encoding prophages, we compared the structures of φSa2 elements carrying *lukS-PV* and *lukF-PV* in 10 ST1232 isolates from Taiwan and six international ST1232 isolates from Korea (BDH17), Japan (THI2018-120, N1195), Australia (S33111-2020, S62531-2020), and Denmark (176761). All isolates harbored highly similar φSa2 prophages, with conserved gene organization and ≥97%–99% sequence identity across the entire region (Fig. 4). These prophages consistently contained genes encoding an integrase, structural components, and PVL toxin genes. Despite being partially assembled in some draft genomes, the complete φSa2 sequences were recoverable and intact, indicating overall genomic stability.

We next examined the φSa3 prophage, which carries the IEC genes *scn*, *chp*, and *sak*. These genes were identified in all 10 Taiwanese ST1232 isolates and in international ST1232 isolates from Korea, Japan, Australia, and Denmark (Fig. S1). The φSa3 elements were highly conserved, exhibiting ≥99% sequence identity and consistent gene architecture across these isolates, suggesting stable chromosomal integration. A comparable φSa3 structure was also detected in three of the four Taiwanese ST398 isolates. One ST398 isolate (SCTW2018065) harbored a slightly divergent φSa3 (96%–98% identity) but retained the complete IEC gene set.

Finally, we analyzed the structure of the *radC*::Tn*554* transposon. This element, integrated at the chromosomal *radC* locus, was identified in seven Taiwanese ST1232 isolates and in representative ST1232 strains from Japan, Korea, Australia, and Denmark. All *radC*::Tn*554*-positive isolates shared an identical structure composed of *tnpA*, *tnpB*, *tnpC*, *ant(9)*, *erm(A)*, and the hypothetical protein genes, with 100% sequence identity across the entire region (Fig. S2). In contrast, this *radC*::Tn*554* was absent in all ST398 isolates.

## DISCUSSION

This study presents the first genomic and phylogenetic characterization of MRSA CC398 isolates in Taiwan, revealing two distinct human-associated lineages: ST1232 and ST398. The ST1232 lineage, which harbored SCC*mec* V(5C2), PVL genes (φSa2), and IEC genes (φSa3), was primarily associated with SSTIs, consistent with previous reports from Japan and Korea (15). In contrast, ST398 lacked PVL and Tn*554*, exhibited greater genetic heterogeneity, and was more frequently isolated from BSIs. Notably, none of the patients had a history of livestock exposure, supporting the possibility of human-to-human transmission within the community.

### Evidence supporting human adaptation of ST1232

All ST1232 isolates in this study carried a stable combination of genetic determinants that are strongly associated with human-adapted *S. aureus* CC398 lineages. These included *lukS/F-PV* (PVL), the IEC (*scn, chp, sak*), and resistance genes, such as *blaZ*, *erm(A)*, and *tet(K)*. In addition, all isolates harbored the transposon Tn*554* integrated at the *radC* locus (*radC*::Tn*554*), comprising *erm(A*), *ant(9)*, and transposition-related genes (*tnpA, tnpB, tnpC*). This conserved integration site has been repeatedly reported among human-associated ST1232 strains in Japan, Korea, and Europe, suggesting early acquisition followed by clonal maintenance (12, 13, 15). The *radC* locus, encoding DNA repair protein C, is known to serve as a common integration hotspot for Tn*554*-family transposons in *S. aureus* (35–37). In our data set, the presence of *radC::*Tn*554* exclusively in ST1232, but not in ST398, highlights this insertion as a stable lineage-associated marker that effectively distinguishes the two closely related CC398 lineages.

Comparative genomic studies further support that ST1232 represents a distinct human-adapted lineage within CC398 (12–15). Reports from Europe, including PVL-positive ST1232 causing human infections in Czech Republic, further highlight its role in international dissemination (13).

SCC*mec* elements in ST1232 were structurally conserved, consistent with clonal expansion, though occasional insertions such as Tn*554* or IS*Sau1* indicate that sporadic acquisitions may continue to shape resistance potential. Together, these findings indicate that ST1232 is both genetically conserved and epidemiologically successful, combining PVL, IEC, and a stable *radC*::Tn*554* integration.

### Genetic diversity and adaptation of ST398

In contrast to the clonally conserved ST1232 lineage, the four Taiwanese ST398 isolates displayed greater genomic heterogeneity. Although PVL-negative, all carried the IEC genes (*scn*, *chp*, *sak*) on φSa3, a hallmark of human adaptation (3, 6, 11). None harbored livestock-associated markers, such as *tet(M)* or *czrC*. Instead, resistance was mediated by *erm(A)* and *tet(K)*, a pattern consistent with previously reported human-associated ST398 (2, 6).

These isolates also exhibited structural diversity in their SCC*mec* elements, including both type V (5C2) and subtype Vb (5C2&5), along with variability in associated mobile elements, such as *ccrC1* alleles, restriction–modification system genes, and insertion sequences. Such heterogeneity reflects the structural plasticity of SCC*mec* and may be driven by ecological adaptation or antimicrobial pressure (2, 3). Therefore, the presence of IEC, the absence of livestock-associated markers, and the lack of livestock contact among patients support that Taiwanese ST398 belongs to a human-adapted lineage. This interpretation is consistent with global reports showing that human-adapted ST398 has re-emerged from human MSSA ancestors and is increasingly responsible for invasive infections in individuals without animal exposure (2, 3, 14). Continued genomic surveillance is warranted to monitor its evolutionary trajectory and assess its potential clinical significance.

### Role of mobile genetic elements in ST1232

The pathogenicity and resistance phenotype of ST1232 are largely shaped by its MGEs. SCC*mec* V (5C2) and Tn*554* confer methicillin and macrolides resistance, while PVL-encoding φSa2 is linked to SSTIs and invasive disease (19, 38). The φSa3 prophage, carrying IEC genes, promotes immune evasion and was consistently found in Taiwanese and international ST1232 isolates (12, 13, 15, 19).

The IEC genes impair complement activation and neutrophil chemotaxis, while PVL lyses neutrophils, contributing to tissue damage (3). Consistent with these mechanisms, a Czech study described PVL-positive CC398 isolates from abscesses in young adults (13), reinforcing the clinical significance of ST1232. In addition, the co-location of *erm(A)* and *ant(9)* within the *radC*::Tn*554* element highlights its role in macrolides and aminoglycoside resistance. The global distribution of this highly conserved *radC*::Tn*554* suggests early acquisition and long-term stability during clonal expansion (13, 15).

The co-localization of virulence and resistance determinants, such as *erm(A)*, *mecA*, and PVL, may provide selective advantages that facilitate clonal expansion and persistence. Potential interactions between φSa3 and other prophages, such as φMR11-like elements, could further enhance virulence under host stress conditions (7). Global reports of frequent co-occurrence of IEC genes and resistance determinants in ST1232 reinforce the lineage’s dual advantage in both antimicrobial resistance and host adaptation (16).

### Global dissemination and comparison with international lineages

Taiwanese ST1232 isolates clustered with strains from Japan, Korea, Australia, and Denmark, confirming shared ancestry and human adaptation (14, 15). A nosocomial outbreak in Denmark was attributed to an ST1232 clone linked to travel from Southeast Asia (8). Similarly, Taiwanese ST398 isolates were closely related to strains from China and Australia, suggesting regional spread or cross-border introduction. ST398 clustering with Chinese strains had already been reported in northern Taiwan as early as 2006, indicating sustained community transmission (18).

Other ST398 strains with similar genotypes have caused serious infections in Australia and China, underscoring their epidemic potential (6). Global phylogenetic analyses position Taiwanese ST398 within the L2–EP4 Asian human-adapted sublineage (16). Collectively, these findings suggest that both ST1232 and ST398 in Taiwan are part of a broader Asia-Pacific CC398 transmission network, emphasizing the need for coordinated international surveillance.

### Public health and clinical implications

ST1232 has been increasingly reported in patients without livestock contact in Asia, Europe, and Australia, consistent with our findings of community-acquired infections in Taiwan (8, 13–15). This lineage’s combination of resistance and virulence determinants raises concern for clinical impact. Infections linked to ST1232 include SSTIs, pneumonia, and bacteremia, while ST398 was associated with three fatal BSI cases in our cohort. These observations highlight the need for improved surveillance. Current systems focusing on LA-MRSA may overlook human-adapted CC398; integrating genomic monitoring and targeted screening of PVL-positive MRSA will be critical to prevent persistent transmission.

### Study limitations and conclusion

This study has several limitations. First, it is based on isolates from only two hospitals in central Taiwan, which may limit generalizability. Second, the absence of functional assays—such as gene expression analysis or *in vivo* models—precludes direct assessment of pathogenic potential. Third, limited epidemiological data hinder our ability to fully elucidate transmission pathways. Future research with broader geographic sampling, functional genomics, and detailed epidemiological investigation will be essential.

In conclusion, our genomic and phylogenetic analyses highlight the emergence of ST1232 as a human-adapted MRSA CC398 lineage in Taiwan, characterized by clonal expansion, conserved MGEs, and phylogenetic similarity to Asian and Australian strains. Although limited in scope, our findings underscore the importance of continued genomic surveillance and tailored infection control strategies. This study provides the first genomic characterization of ST1232 from Taiwan, placing these isolates in the broader context of East Asian and international clonal expansion. While consistent with prior recognition of ST1232 as a human-adapted lineage, our results extend this knowledge by documenting its clinical presence and impact in Taiwan, complementing existing reports from Japan, Korea, and Europe. Finally, while MLST remains useful for lineage classification, WGS offers higher resolution for detecting MGEs and inferring phylogenetic relationships, underpinning our use of cgMLST in this study.

## Data Availability

All raw sequencing data generated in this study have been deposited in the NCBI Sequence Read Archive (SRA) under BioProject accession number PRJNA1234799. Individual sample accession numbers can be found in Table S3. The assembled genomes and associated annotation files are also available under the same BioProject.

## SUPPLEMENTAL MATERIAL

Supplemental material spectrum.02090-25-s0001.docx Tables S1 to S3; Fig S1 and S2.

ASM does not own the copyrights to Supplemental Material that may be linked to, or accessed through, an article. The authors have granted ASM a non-exclusive, world-wide license to publish the Supplemental Material files. Please contact the corresponding author directly for reuse.
