# Supplementary material for: Genomic and phylogenetic characterization of human-adapted methicillin-resistant Staphylococcus aureus clonal complex 398 lineages in Taiwan
Source: Microbiol Spectr. 2025 Dec 23;14(2):e02090-25. doi: 10.1128/spectrum.02090-25 (PMC12889061; doi:10.1128/spectrum.02090-25)
Supplement: Supplemental material — Tables S1 to S3; Fig S1 and S2. [file spectrum.02090-25-s0001.docx]

**Supplemental File**

**Table S1** Reference genomes used in phylogenetic analysis

**Table S2** Tn*554* insertion sites of each ST1232 isolate in this study

**Table S3** Accession numbers in this study

**Supplemental Figure S1** Schematic comparison of φSa3 prophage elements from selected MRSA isolates in Taiwan, Korea, Japan, Australia, and Denmark

**Supplemental Figure S2.** Schematic representation of the Tn*554* transposon identified at the *radC* loci of the ST1232 MRSA isolates

**Table S1** Reference genomes used in phylogenetic analysis

| **Strain** | **Host** | **Country** | **Year** | **Isolation Source** | **MLST** | **Accession & BioSample ID** |
| --- | --- | --- | --- | --- | --- | --- |
| M2009_10003479 | Pig | Hungary | 2009 | not available | ST398 | SAMN00811588 |
| 2007-70-91-4-SPA | Pig | Demark | 2007 | not available | ST398 | SAMN00828678 |
| 9B | Pig | Demark | 2007 | not available | ST398 | SAMEA2493771 |
| PR7/08 | Pig | Portugal | 2007 | dust­ | ST398 | SAMN00828621 |
| S0385 | Human | Netherlands | 2006 | blood | ST398 | [AM990992](https://www.ncbi.nlm.nih.gov/nuccore/AM990992) |
| S51503-2020 | Human | Australia | 2020 | blood | ST398 | SAMN22513566 |
| S42808_2019 | Human | Australia | 2019 | blood | ST398 | SAMN22513567 |
| 51225 | Human | Demark | 2006 | not available | ST398 | SAMN00828686 |
| 55730 | Human | Demark | 2007 | nose | ST398 | SAMN10518803 |
| X05 | Human | China | 2016 | blood | ST398 | SAMN11611372 |
| S31909-2018 | Human | Australia | 2018 | blood | ST398 | SAMN22513574 |
| J12 | Human | China | 2016 | blood | ST398 | SAMEA115538058 |
| R09 | Human | China | 2016 | blood | ST398 | SAMN11611371 |
| J01 | Human | China | 2016 | blood | ST398 | SAMN11611369 |
| RR69 | Human | Taiwan | 2012 | nose | ST1232 | SAMN12257565 |
| S62531-2020 | Human | Australia | 2020 | blood | ST1232 | SAMN22513569 |
| S33111-2017 | Human | Australia | 2017 | blood | ST1232 | SAMN22513570 |
| N1195 | Human | Japan | 2017 | open pus | ST1232 | [NZ_AP024313](https://www.ncbi.nlm.nih.gov/nuccore/NZ_AP024313) |
| THI2018-120 | Human | Japan | 2018 | sputum | ST1232 | [NZ_AP024311](https://www.ncbi.nlm.nih.gov/nuccore/NZ_AP024311) |
| BDH17 | Human | Korea | 2018 | open pus | ST1232 | [NZ_AP024315](https://www.ncbi.nlm.nih.gov/nuccore/NZ_AP024315) |
| 176761 | Human | Denmark | 2013 | skin abscess | ST1232 | SAMN10518807 |

**Table S2** Tn*554* insertion sites of each ST1232 isolate in this study

| **Strain** | **Location** | **Orientation** | **Disrupted Gene^a^** |
| --- | --- | --- | --- |
| CBTW2018008 | 0892957..0899609 | **+** | YwqG family protein |
|  | 1749038..1755690 | **-** | *radC* |
| CBTW2018043 | 0889712..0896364 | **+** | YwqG family protein |
|  | 1745883..1752535 | **-** | *radC* |
|  | 2300073..2306700 | **-** | lactose-specific PTS transporter subunit EIIC |
| CBTW2018311 | 1699375..1706027 | **-** | *radC* |
| CBTW2018367 | 1742868..1749520 | **-** | *radC* |
| SCTW2018482 | 1739148..1745800 | **-** | *radC* |
|  | 2420962..2427614 | **-** | YnfA family protein |
| SCTW2018694 | 1743989..1750641 | **-** | *radC* |
| CBTW20181217 | 0191183..0197835 | **-** | TetR/AcrR family transcriptional regulator |
|  | 1751498..1744846 | **-** | *radC* |
| CBTW2022135 | 0191069..0197721 | **+** | TetR/AcrR family transcriptional regulator |
|  | 1720844..1727485 | **-** | *radC* |
| CBTW2022289 | 0057215..0063864 | **+** | n/a |
|  | 1749314..1755966 | **-** | *radC* |
| SCTW2022336 | 0191192..0197842 | **-** | TetR/AcrR family transcriptional regulator |
|  | 1705750..1712399 | **-** | *radC* |

^a^ Disrupted gene annotations are based on protein predictions generated by PGAP. When available, gene names are provided; otherwise, the corresponding protein family or functional annotation is listed.

**Table S3** Accession numbers in this study

| **Sample Name** | **Accession number** | |
| --- | --- | --- |
|  | **Chromosome** | **Plasmid** |
| CBTW2018008 | [CP184567](https://www.ncbi.nlm.nih.gov/nuccore/CP184567.1) | [CP184568](https://www.ncbi.nlm.nih.gov/nuccore/CP184568.1) |
| CBTW2018043 | [CP184565](https://www.ncbi.nlm.nih.gov/nuccore/CP184565.1) | [CP184566](https://www.ncbi.nlm.nih.gov/nuccore/CP184566.1) |
| SCTW2018065 | [CP184563](https://www.ncbi.nlm.nih.gov/nuccore/CP184563.1) | [CP184564](https://www.ncbi.nlm.nih.gov/nuccore/CP184564.1) |
| CBTW2018311 | [CP184561](https://www.ncbi.nlm.nih.gov/nuccore/CP184561.1) | [CP184562](https://www.ncbi.nlm.nih.gov/nuccore/CP184562.1) |
| CBTW2018367 | [CP184559](https://www.ncbi.nlm.nih.gov/nuccore/CP184559.1) | [CP184560](https://www.ncbi.nlm.nih.gov/nuccore/CP184560.1) |
| SCTW2018482 | [CP184557](https://www.ncbi.nlm.nih.gov/nuccore/CP184557.1) | [CP184558](https://www.ncbi.nlm.nih.gov/nuccore/CP184558.1) |
| SCTW2018694 | [CP184555](https://www.ncbi.nlm.nih.gov/nuccore/CP184555.1) | [CP184556](https://www.ncbi.nlm.nih.gov/nuccore/CP184556.1) |
| CBTW20181217 | [CP184553](https://www.ncbi.nlm.nih.gov/nuccore/CP184553.1) | [CP184554](https://www.ncbi.nlm.nih.gov/nuccore/CP184554.1) |
| SCTW2019099 | [CP184549](https://www.ncbi.nlm.nih.gov/nuccore/CP184549.1) | [CP184550](https://www.ncbi.nlm.nih.gov/nuccore/CP184550.1) |
| SCTW2019145 | [CP184551](https://www.ncbi.nlm.nih.gov/nuccore/CP184551.1) | [CP184552](https://www.ncbi.nlm.nih.gov/nuccore/CP184552.1) |
| SCTW2021015 | [CP184547](https://www.ncbi.nlm.nih.gov/nuccore/CP184547.1) | [CP184548](https://www.ncbi.nlm.nih.gov/nuccore/CP184548.1) |
| CBTW2022135 | [CP184545](https://www.ncbi.nlm.nih.gov/nuccore/CP184545.1) | [CP184546](https://www.ncbi.nlm.nih.gov/nuccore/CP184546.1) |
| CBTW2022289 | [CP184543](https://www.ncbi.nlm.nih.gov/nuccore/CP184543.1) | [CP184544](https://www.ncbi.nlm.nih.gov/nuccore/CP184544.1) |
| SCTW2022336 | [CP184541](https://www.ncbi.nlm.nih.gov/nuccore/CP184541.1) | [CP184542](https://www.ncbi.nlm.nih.gov/nuccore/CP184542.1) |

**Supplemental Figure S1**


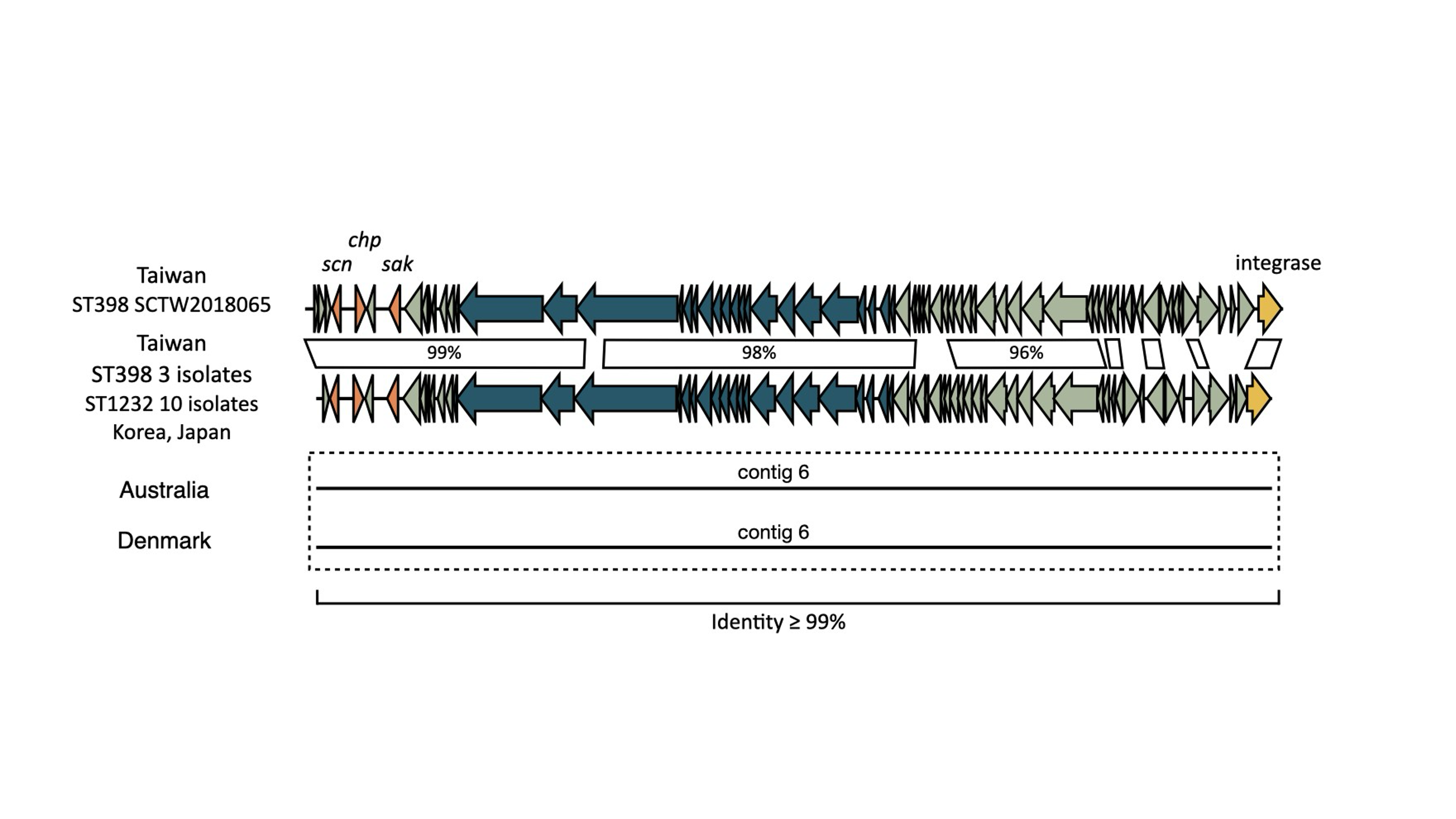


**Supplemental Figure S1.** Schematic comparison of φSa3 prophage elements from selected MRSA isolates in Taiwan, Korea, Japan, Australia, and Denmark. Genes are shown as arrows indicating transcriptional orientation and annotated based on sequence homology. The immune evasion cluster genes (*scn*, *chp*, and *sak*) are specifically marked, and integrase genes are highlighted in yellow. The prophage structures were highly conserved across all ST1232 isolates and four ST398 isolates from Taiwan, with ≥96–99% sequence identity.

**Supplemental Figure S2**


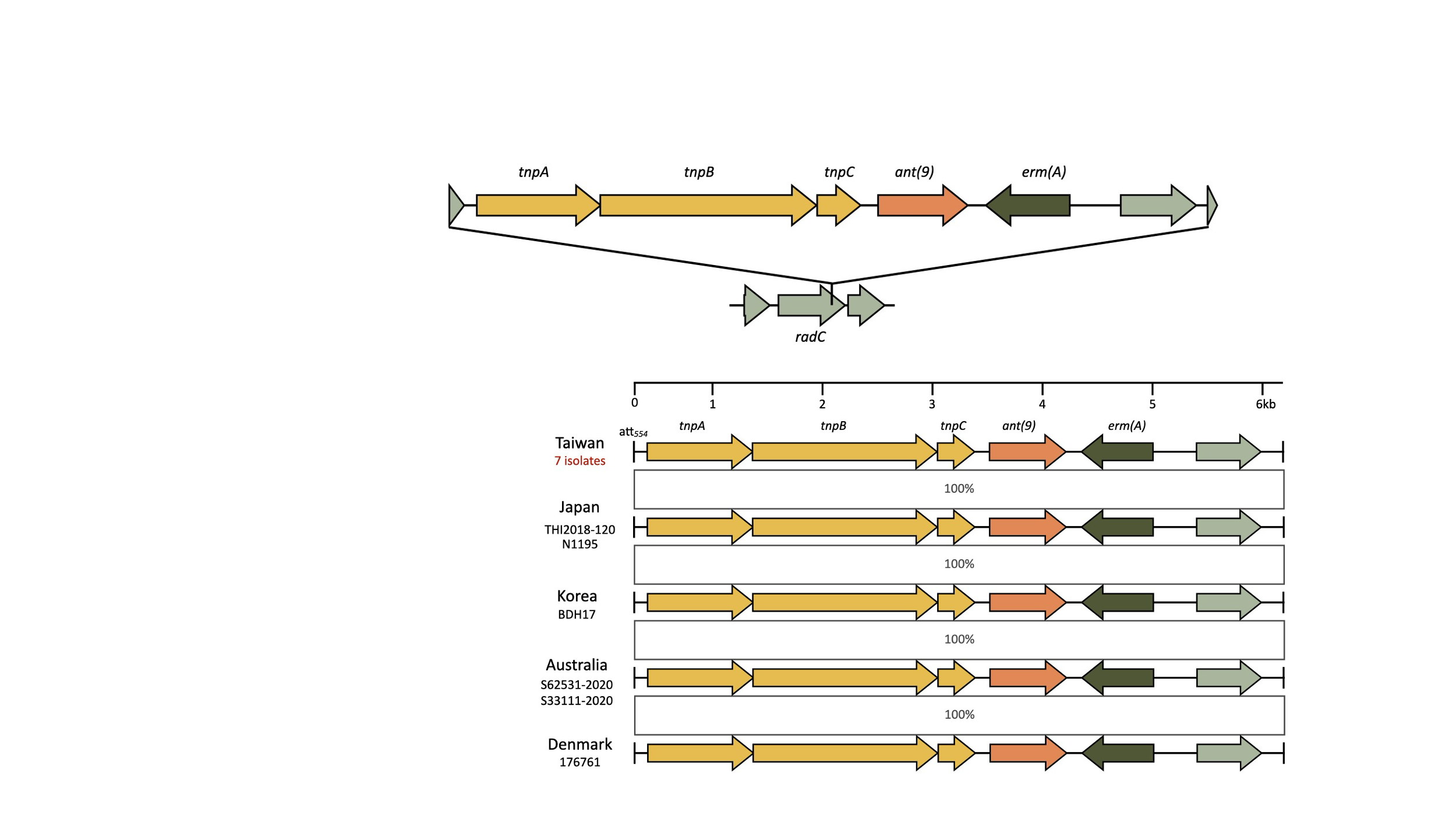


**Supplemental Figure S2.** Schematic representation of the Tn*554* transposon identified at the *radC* loci of the ST1232 MRSA isolates. The element consists of three transposase genes (*tnpA*, *tnpB*, *tnpC*), two resistance genes (*ant(9)* and *erm(A)*), and downstream hypothetical protein genes. This entire ~6-kb region is integrated into the chromosomal *radC* gene, disrupting its open reading frame. Identical *radC*::Tn*554* structures with 100% sequence identity were observed in seven Taiwanese ST1232 isolates and international ST1232 strains from Japan, Korea, Australia, and Denmark. The integration site (*att₅₅₄*) is located immediately upstream of *tnpA*.
